# Growth Performance, Digestive Efficiency, and Meat Quality of Two Commercial Crossbred Rabbits Fed Diets Differing in Energy and Protein Levels

**DOI:** 10.3390/ani12182427

**Published:** 2022-09-15

**Authors:** Marco Birolo, Gerolamo Xiccato, Francesco Bordignon, Sihem Dabbou, Andrea Zuffellato, Angela Trocino

**Affiliations:** 1 Department of Agronomy, Food, Natural Resources, Animal and Environment (DAFNAE), University of Padova, Viale dell’Università 16, 35020 Legnaro, Italy; 2 Center Agriculture Food Environment (C3A), University of Trento, Via E. Mach 1, 38010 San Michele all’Adige, Italy; 3 Research and Innovation Centre, Fondazione Edmund Mach, Via E. Mach 1, 38010 San Michele all’Adige, Italy; 4 A.I.A. Agricola Italiana Alimentare S.p.A., Piazzale Apollinare Veronesi, S. Martino Buon Albergo, 37036 Verona, Italy; 5 Department of Comparative Biomedicine and Food Science (BCA), University of Padova, Viale dell’Università 16, 35020 Legnaro, Italy

**Keywords:** feed efficiency, feed conversion, carcass traits, commercial crossbred

## Abstract

**Simple Summary:**

The nutritional composition of the diets for growing rabbits should be well-balanced, both to meet the requirements of the high-producing hybrids currently used and to avoid nutrient losses. The improvement of the feed conversion ratio represents a key point in increasing rabbit farmers’ profitability because of the high feeding costs. This study evaluated the growth performance, digestive efficiency and meat quality of growing rabbits belonging to the most diffused commercial crossbred rabbit breeds, Grimaud and Hyla, feeding them diets with different energy and protein levels. The two genotypes showed similar growth performance, but Grimaud rabbits achieved higher slaughter yield than Hyla rabbits. The administration of high-energy diets improved feed conversion and increased nutrient digestibility. Conversely, low dietary protein contents reduced nitrogen excretion without negative effects on growth, feed efficiency and carcass traits of fast-growing rabbits.

**Abstract:**

The present study evaluated the effect of digestible energy (DE) and crude protein (CP) levels on growth, digestive efficiency, slaughter yield and meat quality in growing rabbits of two genotypes (Grimaud, G vs. Hyla, H). A total of 384 crossbred rabbits (192 G and 192 H), half males and half females, were divided into eight experimental groups according to a bi-factorial arrangement (2 genotypes × 4 diets; 48 animals/group) and reared in individual cages. From 33 to 64 days of age, rabbits were fed four diets obtained by combining two DE levels (high, HE—10.9 MJ/kg vs. low, LE—9.2 MJ/kg, on average) and two CP levels (high, HP—159 g/kg vs. low, LP—144 g/kg, on average). Then, all rabbits received the same fattening diet (10.7 MJ DE/kg; 156 g CP/kg) until slaughtering (77 days of age). Growth performance did not differ between genotypes for the entire trial (on average final live weight 3010 g; daily weight gain 50.5 g/d), but H rabbits showed a faster growth during the post-weaning period (*p* ≤ 0.01) compared to G rabbits, while exhibiting lower slaughter yield and lower carcass fatness (*p* ≤ 0.01). As DE level increased, feed intake decreased and feed conversion improved (*p* < 0.001), while caecal volatile fatty acid concentration increased and caecal pH and N-ammonia decreased (*p* ≤ 0.01). The reduction in the CP level did not affect performance and carcass traits. No significant interaction was detected between genotype and diet. In conclusion, G rabbits may have an advantage over H rabbits because of the higher slaughter yield. As for the nutritional characteristics of diets for high-producing crossbred rabbits, high energy levels are confirmed to enhance feed efficiency, whereas dietary CP can be reduced to 144 g/kg without negative effects on performance and carcass traits.

## 1. Introduction

During the last two decades, rabbit meat consumption is progressively declining in the European Union (EU) due to several issues, including high production costs, consumers’ concerns for animal welfare (cage-farming systems), unsuitable presentation of the end product (whole carcasses with head), increased popularity of rabbits as pet animals and criticism about the environmental sustainability of rabbit farms [1]. Moreover, despite the fact that rabbit meat is an important product in some member states, especially Spain, France and Italy [2], few breeding companies carry out the genetic selection of pure lines that are then crossed in the farms to obtain the final crossbreds. Genetic suppliers mostly work on national markets (e.g. ANCI and Martini in Italy, Zika in Germany, Sika in Slovenia, Pannon White in Hungary, Universidad Politécnica de Valencia—UPV and Institut of Agrifood Research and Technology—IRTA in Spain), while French genetic lines (Hypharm-Eurolap and Hycole) are widely spread in EU [3]. Nonetheless, few studies have compared the main French genotypes in terms of growth performance, slaughter results and carcass traits [4,5].

Genetic selection, feeding and nutritional strategies aimed at optimising growth rate and feed efficiency are the main tools to reduce feed conversion ratio (FCR) and enhance farmer profitability [6,7]. In fact, in intensive rabbit farms, the variable costs (e.g. feeding, replacement and artificial insemination) impact more than 60–65% of the total costs, and the feeding of growing rabbits represents the major expenditure [8,9]. In the fattening stage, FCR improves when reducing feed intake without changes in daily weight gain and/or when reducing morbidity and mortality rates [7]. In rabbits fed ad libitum, the dietary digestible energy (DE) content explains a large part of the variability of feed intake and FCR [10]: an increase by 1 MJ/kg in dietary DE is expected to reduce the FCR by 0.29–0.40 units [11]. Therefore, the use of high-DE diets is recommended for rabbits during the late fattening phase [12], when the FCR becomes rapidly impaired as a consequence of the decreased growth rate and increased maintenance requirements and fat deposition [7]. By contrast, low-energy high-fibrous diets are generally used during the post-weaning period with the aim of reducing morbidity and mortality related to digestive disturbances [13,14,15]. However, if sufficient levels of lignocellulose (acid detergent fibre, ADF ≥ 180 g/kg and lignin > 50 g/kg) are guaranteed, the DE content can also be increased in post-weaning diets, with further benefits for rabbit feed efficiency [16,17]; this can be achieved either by the simultaneous increase in starch and soluble fibre [18,19], or by replacing starch with fat [12]. 

As for protein requirements in growing rabbits, they are age dependent, being higher in the first post-weaning phase as a consequence of the rapid tissue accretion, gut development, and the need for maintaining intestinal mucosa functionality [20]. However, despite the minimum dietary CP content of 157 g/kg required in young rabbits before the 7th−8th week of age [21], with sufficient essential amino acid supplementation, low-protein diets (140 g CP/kg) can be administered during the post-weaning and fattening period without negative effects on rabbit growth [22]. Furthermore, the decrease in dietary CP has been associated with reduced morbidity and mortality rates, as well as reduced nitrogen excretion in rabbit farms [21,23,24,25]. On the other hand, recent studies have hypothesised that moderate CP diets (146 g/kg), despite being supplemented with synthetic amino acids, might not cover the protein requirements of fast-growing rabbits [26]. Such a nutritional deficit could be accentuated with high-DE diets, which significantly reduce feed consumption, and thus the protein and essential amino acid intake.

Moreover, both growth performance and carcass composition could be more or less affected by changes in DE and CP levels depending on the genotype, with fast-growing rabbits especially affected, and on the animals’ resilience in the presence of dietary nutritional changes.

Therefore, the present study aimed at evaluating the effects of the levels of dietary DE and CP on growth performance, diet digestibility, caecal fermentation activity and carcass and meat quality traits in fast-growing rabbits of the two commercial crossbred lines of French origin most commonly reared in France and in Italy.

## 2. Materials and Methods

### 2.1. Animals and Experimental Conditions

The trial was realised at the experimental farm “L. Toniolo” of the University of Padova, in a brick shed and under a natural photoperiod during the months of April and May. Extraction fans and an automatic heating system were used to control air circulation and relative humidity and to maintain the temperature within 17–24 °C.

A total of 384 crossbred rabbits: 192 Grimaud (female PS Hyplus Optima × male PS 59; Hypharm, Groupe Grimaud, Roussay, France) and 192 Hyla (female PS Hyla × male Hyla Max; Eurolap, Sevremoine, France), with half being females and half males, were selected on two commercial farms (one farm per genotype). On both farms the rabbit does were synchronised and inseminated on the same day, 18 days post-partum, and housed in multifunctional cages. Rabbits were selected from healthy litters (standardised at 9 kits per doe) of multiparous does (3 to 4 kindling) and moved the same day to the experimental facilities at 33 days of age. Rabbits were individually identified using ear marks, housed in individual cages (285 mm × 410 mm × 285 mm) and randomly allocated to eight experimental groups (48 animals per group; half females and half males), homogeneous by average live weight and standard deviation, according to a bi-factorial arrangement with two genotypes (G: Grimaud vs. H: Hyla) and four experimental post-weaning diets obtained from the combination of two DE levels (HE: high DE—10.9 MJ/kg vs. LE: low DE—9.2 MJ/kg, on average) and two CP levels (HP: high CP—159 g/kg vs. LP: low CP—144 g/kg, on average).

### 2.2. Experimental Diets

The ingredients and chemical compositions of the experimental diets are listed in Table 1.

The post weaning diets were administered from 33 to 64 d of age. The increase in DE from LE diets to the HE diets was realised by increasing the inclusion rates of barley (+155 g/kg on average), soybean oil (+10 g/kg on average), soybean meal (+60 g/kg on average) and dried beet pulp (+95 g/kg on average) at the expense of dehydrated alfalfa meal (−234 g/kg on average), wheat bran (−50 g/kg on average) and sunflower meal (−35 g/kg on average). This way, the HE diets were characterised by higher contents of lipids (+7 g/kg on average) and starch (+44 g/kg on average) and lower contents of neutral detergent fibre (aNDF; −67 g/kg on average), acid detergent fibre (ADF; −56 g/kg on average) and lignin (sa) (−18 g/kg on average) with respect to LE diets. The higher CP level of HP diets compared to LP ones was related to the partial or total substitution of the low-CP dehydrated alfalfa meal (14.3 g CP/100 g) with the high-CP dehydrated alfalfa meal (16.5 g CP/100 g), besides a higher inclusion rate of soybean and/or sunflower meals.

Due to the presence of enteric diseases in the commercial farms where the rabbits were born, all post-weaning diets (fed from 33 to 64 days of age) were supplemented with an antibiotic (a.p. oxytetracycline, 1450 mg/kg; administered by inclusion in feed mixture preparation of 7250 mg/kg diet of OXITER 200 BMP—200 mg oxytetracyclin/g—DOX-AL ITALIA S.p.A., Milan, Italy), besides the standard coccidiostat supplementation (p.a. diclazuril, 1 mg/kg; obtained by the dietary inclusion of 0.5 g/kg diet of Coxirill® 0.2%—Huvepharma, Sofia, Bulgaria).

From 65 days of age until slaughtering (77 days of age), a unique fattening diet (diet F) without antibiotics and coccidiostats was provided to all rabbits (Table 1). 

The pelleted diets (diameter: 3.5 mm; length: 10–11 mm) were supplemented with synthetic lysine and methionine, vitamins and macro- and micro-minerals to satisfy the nutritional requirements of rabbits during the post-weaning and fattening periods [27]. All animals had ad libitum access to feed and water during the whole trial.

### 2.3. Recordings and Health Monitoring

During the trial, the individual live weight and feed intake of the animals were recorded once a week from the arrival of the rabbits until the day before commercial slaughtering. The health of the animals was monitored daily: rabbits were considered ill when showing diarrhoea and/or mucus in the faeces or a live weight loss during a week.

### 2.4. Digestibility Trials

The nutrients’ apparent digestibility and the nutritive value of the diets were measured during two in vivo digestibility trials performed according to the European standardised method [28]. The first trial was carried out on 72 rabbits out of those on trial (36 rabbits per genotype; 18 per diet; 9 per experimental group) from 46 to 50 days of age, when the animals were fed with the four post-weaning diets. The second trial was performed on 32 rabbits (16 rabbits per genotype) from 72 to 76 days of age, when the rabbits were fed diet F.

### 2.5. Sampling of Caecal Content

At 50 days of age, 48 rabbits (24 rabbits per genotype; 12 per diet; 6 per experimental group), half of them females and half males, representative of the corresponding experimental groups in terms of mean live weight and standard deviation, were slaughtered to allow sampling of their caecal content in the afternoon (between 13:30 and 15:10).

Rabbits were weighed immediately before slaughtering and killed by cervical dislocation. Stomach and gut were removed and weighed. The caecum was removed and weighed, and the pH of the caecal content was immediately measured. The caecal content was then diluted with a 15% HPO_3_ solution (25% wt/wt) and stored at −20 °C until the chemical analyses.

### 2.6. Commercial Slaughtering and Carcass and Meat Quality Recordings

At 76 days of age, the rabbits were weighed without previous fasting to determine the final live weight and to measure growth performance. At 77 days of age, the rabbits on trial were weighed at the experimental farm before loading and after a 4 h fasting period, as required by the slaughterhouse. All healthy rabbits with a live weight exceeding the minimum weight of 2,300 g required by the slaughterhouse (323 overall; 159 Grimaud and 164 Hyla; 82 from diet HE–HP, 82 from diet LE–HP, 79 from diet LE–HP and 80 from diet LE–LP) were properly caged and transported to a commercial slaughterhouse by an authorised truck.

The number of rabbits discarded by trial due to illness and/or insufficient live weight was used to calculate the total losses rate as follows:

$$\mathrm{Total}\;\mathrm{losses}\;\mathrm{rate}\;\left(\%\right)=\frac{\left[\mathrm{dead}\;\mathrm{rabbits}\;\left(n\right)+\mathrm{discarded}\;\mathrm{rabbits}\;\left(n\right)\right]}{\mathrm{initial}\;\mathrm{rabbits}\;\left(n\right)}\times 100$$

Rabbits slaughtered for sampling of the caecal content were not included in the initial number of animals.

The travel from the experimental farm to the commercial slaughterhouse lasted about 1 h. Slaughtering took place about 1 h after the animals arrived at the slaughterhouse, where they were individually weighed, stunned by electro-anesthesia, and killed by jugulating. After 2.5 h of chilling, the commercial carcasses were weighed to calculate the individual slaughter yield [29], and the feed-to-meat conversion ratio according to the following formula:

$$\mathrm{Feed}\;\mathrm{to}\;\mathrm{meat}\;\mathrm{conversion}\;\mathrm{ratio}=\frac{\mathrm{total}\;\mathrm{feed}\;\mathrm{intake}\;\left(g\right)}{\mathrm{chilled}\;\mathrm{carcass}\;\mathrm{weight}\;\left(g\right)}$$

The full gastrointestinal tract weight was recorded individually and expressed as a percentage of the slaughter.

Moreover, the proportion of other slaughter wastes (skin, distal part of fore and hind legs and blood) was calculated as the difference between slaughter weight, full gastrointestinal tract weight and carcass weight and expressed as a percentage of slaughter weight according to the the following formula:

$$\mathrm{Other}\;\mathrm{slaughter}\;\mathrm{wastes}\;\left(\%\right)=\frac{\left[\mathrm{slaughter}\;\mathrm{weight}\;\left(g\right)-\mathrm{full}\;\mathrm{gastrointestinal}\;\mathrm{tract}\;\left(g\right)-\;\mathrm{chilled}\;\mathrm{carcass}\;\mathrm{weight}\;\left(g\right)\right]}{\mathrm{slaughter}\;\mathrm{weight}\;\left(g\right)}\times 100$$

A total of 160 carcasses (80 per genotype; 40 per diet, half females and half males), representative of the corresponding experimental groups in terms of average rabbit live weight and standard deviation, were selected, transported to the department laboratory and stored at 3–4 °C for 24 h. Then, the reference carcass and the main cuts for meat quality analyses were obtained according to harmonised dissection procedures described by Blasco and Ouhayon [29].

After that, the pH value of the right *l. lumborum* muscle was measured in duplicate using a pH meter (Basic 20; Crison Strumenti Spa, Carpi, Italy) equipped with a specific electrode (cat. 5232). Duplicate measurements of the L*a*b* colour indexes [30] were obtained from the same muscle using a Minolta CM–508 C spectrophotometer (Minolta Corp., Ramsey, NJ, USA). The hind legs and *l. lumborum* muscles were dissected and the meat-to-bone ratio of the hind legs was measured [29].

The right *l. lumborum* muscles were stored at −18 °C in vacuum-sealed plastic bags until determination of thawing and cooking losses and of shear force. In detail, after thawing for 12 h at room temperature, the whole muscles were weighed to measure thawing loss, sealed in plastic bags, and cooked in a water bath for 1 h at 80 °C. After a 1 h cooling period at room temperature, the *l. lumborum* were weighed to measure cooking losses, and a cut from the middle part was separated (length: 70 mm). On this section, the maximum shear force was measured by means of a TA.HDI dynamometer (LS5; Lloyd Instruments Ltd., Bognor Regis, UK) with the Allo–Kramer (10 blades) probe (load cell: 500 kg, distance between the blades: 5 mm, thickness of blades: 2 mm and cutting speed: 500 mm/min).

### 2.7. Chemical Analyses

The diets and faeces were analysed to determine the contents of dry matter (934.01), ash (967.05), crude protein (2001.11) and starch (amyloglucosidase α amylase method, 996.11) using AOAC [31] methods. Ether extract was determined after acid hydrolysis [32]. The neutral detergent fibre (aNDF) was analysed according to Mertens [33], assayed with a heat stable amylase without sodium sulphite and expressed inclusive of residual ash. The acid detergent fibre (ADF), expressed inclusive of residual ash, was assessed according to AOAC ([31], method 973.187). The lignin (determined by solubilisation of cellulose with sulphuric acid) was analysed according to Van Soest et al. [34]. The sequential procedure and the filter bag system (Ankom Technology, Macedon, NY, USA) were used in the analyses. Gross energy contents of diets and faeces were measured by an adiabatic bomb calorimeter (IKAC200, Staufen, Germany). 

The thawed samples of caecal contents were centrifuged at 9000 rpm for 10 min. Caecal N ammonia was determined on the supernatant with a pH meter (GLP 22, Crison Strumenti Spa) equipped with ammonia specific electrode (mod. 9663 combined with the reference electrode mod. 5044). Volatile fatty acid (VFA) molar contents were measured on the supernatant with gas chromatography (Agilent 7820A, Agilent Santa Clara, CA, USA) equipped with a flame ionisation detector FID split–splitless injection system programmable oven on a cross bond capillary column DB–FFAP (30 m × 0.25 mm I.D., 0.25 μm film thickness) (Agilent) according to the method described by Osl [35].

### 2.8. Statistical Analyses

The data were analysed by a two–way ANOVA using the PROC GLM of SAS 9.4 software (SAS Institute Inc., Cary, NC, USA) [36]. The model included the genotype and the diet and their interactions as fixed effects, and the weaning weight as covariate. Contrast statements were used to compare DE energy content and CP level:

$$\mathrm{DE}=\left(\frac{\;\left(\mathrm{HE}–\mathrm{HP}\;+\;\mathrm{HE}–\mathrm{LP}\right)}{2}\right)-\left(\frac{\;\left(\mathrm{LE}–\mathrm{HP}\;+\;\mathrm{LE}–\mathrm{LP}\right)}{2}\right)\;\mathrm{DE}=\left(\;\frac{\left(\mathrm{HE}–\mathrm{HP}\;+\;\mathrm{LE}–\mathrm{HP}\right)}{2}\right)-\left(\frac{\;\left(\mathrm{HE}–\mathrm{LP}\;+\;\mathrm{LE}–\mathrm{LP}\right)}{2}\right)$$

Overall, differences among means with a *p*-value < 0.05 were accepted as representing statistically significant differences. Least-square means were compared using the Bonferroni test.

## 3. Results

### 3.1. Health and Growth Performance

During the trial, six rabbits died (one from group G/HE–HP, three from group G/LE–HP, one from group H/HE–LP and one from group H/LE–HP) showing diarrhoea and/or mucoid enteritis. At the end of the trial, another seven rabbits (one from group G/HE–HP, one from group G/HE–LP, three from group G/LE–LP, one from group H/LE–HP and one from group H/LE–LP) were excluded from the trial and the commercial slaughtering due to the low live weight (<2300 g). The mortality rate in the whole trial was 1.8%, and the total losses rate reached 3.9%, without significant differences among groups (data not reported in tables).

The initial live weight significantly differed between G and H rabbits (*p* < 0.001); thus, it was used as covariate in the analysis of growth performance, feed intake and FCR. The covariate was significant for live weight at 64 and 76 days of age, and for feed intake and FCR in the post-weaning, fattening and whole trial periods. The H rabbits were heavier than the G rabbits at weaning (+53 g; *p* < 0.001) and at 64 days of age (+52 g; *p* = 0.006; Table 2). Compared to G rabbits, H rabbits showed a higher daily weight gain (+1.7 g/d; *p* = 0.006) and feed intake (+4 g/d; *p* = 0.024) during the post-weaning period, whereas they grew and ate less during fattening (−2.7 g/d weight gain; *p* = 0.004; −8 g/d feed intake; *p* = 0.001).

The increase in the DE content reduced feed intake during post-weaning (−23 g/d on average; *p* < 0.001), fattening (−13 g/d on average; *p* < 0.001) and the whole trial (−20 g/d on average; *p* < 0.001). Rabbits fed LE diets during post-weaning period exhibited a higher daily weight gain during fattening compared to rabbits fed HE diets (+5.8 g/d on average; *p* < 0.001), whereas no significant difference was observed in the post-weaning or in the whole trial periods. The rabbits fed LE diets also showed a worse FCR compared to those fed HE diets from 37 to 64 days of age (+0.51 units; *p* < 0.001), whereas an opposite trend was observed from 64 to 76 days of age (−0.23 units; *p* = 0.001), when all rabbits received a unique fattening diet. Through the whole trial, the use of HE diets in the post-weaning period improved FCR compared to LE diets (−0.35 units; *p* < 0.001).

The dietary protein level did not affect growth, feed intake or FCR of the rabbits. However, in the post-weaning period, a worse FCR was observed in rabbits fed an LE–HP diet (*p* < 0.001). No significant interaction was observed between genotypes and diets with respect to rabbit growth performance.

### 3.2. Digestibility and Nutritive Value of Experimental Diets

As for the genotype, the apparent digestibility of CP was higher in G than H rabbits (+0.9 percentage units; *p* < 0.001), whereas the digestibility of the ether extract was higher in H than G rabbits (+2.0 percentage units; *p* < 0.001; Table 3).

As DE content increased in diets HE compared to diets LE, the apparent digestibility of dry matter (+17.8%), CP (+3.3%), ether extract (+6.0%), gross energy (+19.2%) and all fibre fractions (*p* < 0.001) increased, whereas the digestibility of starch decreased (−0.9%; *p* < 0.001; Table 3).

The CP level did not affect the nutrient apparent digestibility of the diets. However, the digestibility of dry matter and gross energy reached the lowest value when low energy levels were associated with high CP levels (*p* < 0.001). The combination between DE and CP levels led to a DP to DE ratio varying from 10.5 g/MJ (HE–LP diet) to 11.3 g/MJ (HE–HP diet) to 11.5 g/MJ (LE–LP diet) to 13.1 g/MJ (LE–HP diet).

The apparent digestibility of diet F, administered in the fattening period, was slightly affected by the genotype (Table 4). In detail, the digestibility of CP (*p* = 0.045) and ether extract (*p* < 0.001) was higher in H rabbits compared to G rabbits. As for the residual effect of post-weaning diets, a lower digestibility of ether extract was observed in rabbits previously fed HE diets (*p* < 0.05).

### 3.3. Caecal Fermentation Traits

At 50 days of age, the genotype did not affect full-gut and full-caecum proportions or caecal fermentative activity (Table 5). However, the full-stomach proportion was higher in H than G rabbits (+7.0%; *p* = 0.034).

The increase in DE content decreased caecal pH (−0.18 units; *p* = 0.006) and N ammonia content (−35.7%; *p* = 0.026) in rabbits fed HE diets compared to those fed LE diets, besides increasing total VFA concentration (+17.4%; *p* = 0.005).

### 3.4. Carcass and Meat Quality Traits

As for the effect of genotype on carcass traits, G rabbits achieved a higher carcass yield compared to H rabbits (+2.0 percentage units; *p* < 0.001) due to a higher chilled carcass weight (+55 g; *p* < 0.001) and lower incidence of full gastrointestinal tract (−1.6 percentage units; *p* < 0.001) and other slaughter wastes (ears, skin, distal part of fore and hind legs, blood; *p* = 0.001) (Table 6). Moreover, G rabbits exhibited a heavier reference carcass (+42 g; *p* = 0.020) and a lower proportion of dissectible fat (−0.38 percentage units; *p* = 0.006); finally, they showed a feed-to-carcass conversion ratio (feed intake/chilled carcass weight) lower than that of H rabbits (3.73 vs. 3.84; *p* < 0.001; data not reported in tables). Regarding meat quality traits, only the lightness index was higher in H than G rabbits (*p* = 0.010).

When rabbits were fed diets with increased DE content, in the post weaning period, transport losses (−0.32 percentage units; *p* = 0.012) and the proportion of hind leg (−0.45 percentage units; *p* = 0.018) decreased while slaughter wastes increased (+0.5 percentage units; *p* = 0.005). The feed-to-carcass conversion ratio increased in LE diets, especially when combined with a high crude protein level (3.58 vs. 3.58 vs. 4.06 vs. 3.95 for the diets HE–HP, HE–LP, LE–HP and LE–LP, respectively; *p* < 0.001; data not reported in table).

## 4. Discussion

The present study was designed to evaluate the animal response in terms of growth performance, slaughter results and carcass and meat quality characteristics of rabbits belonging to two widespread French crossbred genotypes fed diets differing in energy and protein levels. Since no significant genotype × diet interaction was detected in the present trial, the results are discussed separately for the main effects.

### 4.1. Effect of Genotype

Rabbits of the two tested genotypes showed different live weights at weaning, which could be related to different reproductive (litter size) and productive (milk yield) performances of the does [37,38,39]. However, Grimaud and Hyla rabbits came from two different commercial farms, and many factors, including management, diets provided to lactating does and environmental and hygienic conditions of the farms could have affected the weaning weight of the animals. Since a higher weaning weight has been associated with higher feed intake and weight gain in growing rabbits [40,41], the weaning weight was used as covariate for the analysis of growth performance and carcass traits in the present trial.

According to our results, Hyla rabbits seemed to be more precocious than Grimaud rabbits and able to achieve a suitable slaughter weight earlier (2500–2700 g depending on the market). Indeed, during the post-weaning period, Hyla rabbits grew more than Grimaud rabbits, reaching a higher body weight at 64 days of age. The higher growth rate was strictly correlated to increased feed consumption, as observed by other authors when comparing rabbits from paternal lines (selected for growth rate) with rabbits from maternal lines (selected for litter size) [42,43,44]. Despite the differences in live weight at 64 days of age, both genotypes reached similar growth performance and a comparable body weight at slaughtering (2987 g on average at 77 days of age). In fact, both commercial hybrids are obtained by a three-way cross among pure lines with an adult weight ranging from 4000 g to 5000 g; thus, different genotypes tend to reach a similar slaughter weight and maturity degree when slaughtered between 11 and 13 weeks of age [45,46]. However, in the present trial, Hyla rabbits showed a lower carcass weight and a substantially lower carcass yield compared to Grimaud rabbits. When slaughtered at the same age (60 or 67 days of age) or at fixed live weight (1800, 2050 and 2300 g), fast-growing rabbits (from paternal lines) achieve higher body weight compared to slow-growing rabbits (from maternal lines), but with a decrease in carcass yield due to a lower degree of maturity [47,48,49]. Therefore, we can hypothesise that the lower carcass weight and yield detected in Hyla rabbits at 77 days of age could be exacerbated in the case of earlier slaughtering (65–70 days).

The different carcass yield and main cut proportions between genotypes are explained by different allometric coefficients of body tissues and organs. Indeed, depending on the selection program, rabbits of different genotypes could exhibit some differences in the relative growth of body tissues [50]. The increased proportion of full gastrointestinal tract in Hyla rabbits can be related to their higher feed intake in the first growth period, when the development of internal organ is relatively high [51]. Otherwise, Grimaud rabbits eat and grow more during the second period, when the development of late maturing parts of the body increases, such as for forelegs, loin and hind legs muscles [5]. Moreover, Martínez-Bas et al. [5] reported that Grimaud rabbits present an earlier growth of loin compared to rabbits belonging to a Hyla maternal line, whereas the latter were characterised by an earlier development of fat depots, as also observed in the present trial.

Differences in the relative growth of body tissues could be responsible for changes in feed efficiency. In this regard, Szendrő et al. [52] stated that rabbits with higher muscle mass and lower body fat content consume less feed for the same weight gain because the energy demand for muscle development is lower compared to that for fat. In our study, the genotype had no effect on FCR through the whole trial period, but Hyla rabbits showed a higher proportion of dissectible fat and a higher (worse) feed-to-carcass conversion ratio compared to Grimaud. In rabbit does, the selection for reproduction criteria was proven to increase the digestive utilisation of nutrients [53]. In pigs, the selection for lean growth efficiency was associated with a higher protein digestibility and nitrogen retention [54]. In the present study, the apparent digestibility of crude protein was higher in Grimaud than Hyla rabbits during the post-weaning period, whereas an opposite trend was observed during fattening. Contrarily, the apparent digestibility of ether extract was greater in Hyla rabbits during both the post-weaning and fattening periods, which could have contributed to the increase in body fat depots in this genotype.

### 4.2. Effect of Dietary Digestible Energy and Crude Protein Levels

The use of low-energy diets (rich in insoluble fibre) is generally recommended in growing rabbits during the post-weaning period to reduce morbidity and mortality rates due to digestive diseases [14,16,17]. Otherwise, high-energy diets, obtained by increasing the content of highly digestible carbohydrates at the expense of dietary fibre or by partially replacing cereals with fat, are recommended during the last fattening phase to reduce feed intake, improve FCR and minimise mineral excretion [7,11,12]. 

In this study, a standard feeding program (low-energy diets during post-weaning and high-energy diets during the fattening period) was implemented as a control group in the form of an LE diet (9.2 MJ DE/kg) in the first four weeks after weaning and a high-energy diet (10.7 MJ DE/kg) during the last twelve days of fattening. From the other side, in the HE groups, rabbits were fed high-energy diets throughout the rearing period (10.9 MJ DE/kg during post-weaning and 10.7 MJ DE/kg during fattening). The increase in DE level significantly reduced feed intake and FCR, in agreement with previous studies [55,56,57]. Indeed, rabbits regulate their feed intake according to the digestible energy content of diets to satisfy their requirements [10]; thus, when DE increases, FCR improves, without effects on growth [58,59], as observed in the present trial. In other studies, the increase in DE from low (9.1–9.6 MJ/kg) to high levels (10.1–10.4 MJ/kg) also improved the daily weight gain of rabbits fed ad libitum or submitted to feed restrictions [55,56]. In the present trial, after the change to a fattening (more concentrated) diet, LE rabbits maintained a higher feed consumption compared to HE rabbits, thus achieving an increased DE intake and a higher growth rate in the last rearing phase.

Both in the present trail and in other studies, the use of high-energy diets increased rabbit digestive efficiency [55,59] and modified the caecal environment [60]. The inclusion of vegetable oil and other raw materials rich in high-digestible substrates, such as barley, soybean meal and sugar beet pulps, at the expense of alfalfa meal, explains the higher digestibility of HE compared to LE diets [61,62].

The caecal fermentation traits changed significantly with the increase in DE level as a consequence of the greater availability of highly-fermentable carbohydrates, which promotes a higher production of VFA and reduces pH and ammonia nitrogen levels [62,63]. In fact, caecal VFA concentration is positively correlated with increasing levels of low-lignified fibers, which are more susceptible to microflora fermentation in the caecum [64].

As dietary protein level is concerned, a CP content ranging from 150 to 160 g/kg is recommended in the diets of growing rabbits [27]. However, in recent years, the use of low-protein diets (about 140 g/kg) has become common in practical conditions to reduce the risk of digestive diseases [20] and limit nitrogen excretion [23]. Indeed, high CP levels could increase the nitrogen flow reaching the ileum, favour the proliferation of potentially pathogenic bacteria and increase mortality rates [65]. From the other side, in good health conditions, excessive amounts of dietary protein are used for energy purposes through a metabolic pathway that leads to increased urea excretion [21]. Moreover, several studies have shown that a decrease in dietary CP from high (~170 g/kg) to moderate (~150 g/kg) or from moderate to low levels (~140 g/kg) does not affect rabbit growth performance [19,22,66]. More recently, Marín-García et al. [67] confirmed that a diet with a moderate CP content (150 g/kg) supplemented with essential amino acids according to the current recommendations can satisfy the growth requirements of rabbits from a paternal line with a high growth rate (54 g/d on average). Similarly, in the present trial, the decrease in CP level from 159 to 144 g/kg had no effect on the growth performance of fast-growing crossbred rabbits during the post-weaning period or through the whole trial periods. Only when the CP was reduced from very high (>170 g/kg) to very low levels (<140 g/kg) was a significant decrease in weight gain observed in the post-weaning [24] period, or even through the whole fattening period [21,63].

With regard to fermentative activity, a decrease in dietary CP has been associated with decreased total VFA concentration [63,66] and increased pH [59], likely due to a lowered flow and availability of nitrogen for microbial activity [68]. However, other authors did not observe significant variations in the caecal fermentation traits according to the dietary CP content [69], which is consistent with the findings of the present study. The type of nutrient used in substitution of CP, the types and characteristics of dietary fiber (degree of lignification and solubility) and the particle size of the diet may account for the different results among studies [64].

In the present trial, the association of high CP level with low DE concentration decreased the digestibility of dry matter and gross energy and worsened the FCR and the feed-to-carcass conversion ratio, confirming that excessive DP to DE ratio reduces the efficiency of protein utilisation [25]. The feed-to-carcass conversion ratio is widely used as an efficiency indicator by the feed industry when it controls the whole rabbit production chain, from rabbit feed production to meat marketing; it considers the effective feed cost needed to produce 1 kg of marketable carcass, excluding the losses at slaughterhouse. Moreover, the higher proportion of sunflower meal (rich in low-soluble fibre) at the expense of dried beet pulp (rich in high-soluble fibre) in the LE–HP diet compared to the LE–LP diet could partially explain the impaired diet digestibility and feed efficiency [16,18].

As reported in the current study, several authors observed that once the energy and protein requirements were met, further increases in the dietary DE and CP contents weakly affected the slaughter results and meat quality traits of growing rabbits [46].

## 5. Conclusions

In the conditions of the present trial, Grimaud and Hyla rabbits achieved similar growth performance and final live weights, but the former breed showed higher carcass yield and feed-to-carcass conversion ratio.

The increase in DE content to 10.9 MJ/kg in the post-weaning diets, once minimal insoluble fibre contents are guaranteed, can improve feed conversion ratio and reduce feeding costs. Moreover, when diets are supplemented with essential amino acids according to the current recommendations, the dietary CP level can be reduced to about 140 g/kg without affecting the growth and feed efficiency of high-producing fattening rabbits.

## Figures and Tables

**Table 1:** Ingredients and chemical composition (g/kg as fed) of the experimental diets.

	Post-Weaning Diets (33–64 days)				Fattening Diet (64–77 days)
	HE–HP	HE–LP	LE–HP	LE–LP	F
Ingredients					
Dehydrated alfalfa meal (16.5 g CP/100 g)	200.0	117.0	155.0	0.0	180
Dehydrated alfalfa meal (14.3 g CP/100 g)	100.0	170.0	360.0	503.3	120
Wheat bran	200.0	260.0	240.0	320.0	225
Barley meal	150.0	160.0	0.0	0.0	160
Dried sugar beet pulp	180.0	210.0	80.0	120.0	200
Soybean meal (49 g CP/100 g)	80.0	40.0	0.0	0.0	70
Sunflower meal (30 g CP/100 g)	50.0	0.0	140.0	30.0	0.0
Soybean oil	10.0	10.0	0.0	0.0	15
Cane + sugarbeet molasses	15.0	15.0	15.0	15.0	15
Calcium carbonate	2.0	1.5	0.0	0.0	1.0
Dicalcium phosphate	4.0	5.0	0.0	0.0	4.5
Marine salt	4.0	4.0	4.0	4.0	4.0
L–lysine HCl (70 g lysine/100 g)	0.0	1.5	1.0	2.0	0.5
DL–methionine (100 g methionine/100 g)	0.5	1.5	0.5	1.2	1.0
Vitamin–mineral premix ^1^	4.0	4.0	4.0	4.0	4.0
Coccidiostat ^2^	0.5	0.5	0.5	0.5	0.0
Oxytetracycline (mg/kg) ^3^	1450	1450	1450	1450	0.0
Chemical composition					
Dry matter	892	889	898	895	885
Crude protein	159	146	159	142	156
Ether extract	32	34	24	27	43
Ash	71	68	74	67	75
Neutral detergent fibre (aNDF)	345	345	415	408	337
Acid detergent fibre (ADF)	195	192	257	242	186
Lignin (sa)	50	47	69	63	45
Starch	122	134	75	93	136

^1^ Premix provided per kg of complete diet: vit. A, 12000 UI; vit. D3, 1000 UI; vit. E acetate, 50 mg; vit. K3, 2 mg; biotin, 0.1 mg; thiamine, 2 mg; riboflavin, 4 mg; vit. B6, 2 mg; vit. B12, 0.1 mg; niacin, 40 mg; pantothenic acid, 12 mg; folic acid, 1 mg; Fe, 100 mg; Cu, 20 mg; Mn, 50 mg; Co, 2 mg; I, 1 mg; Zn, 100 mg; Se, 0.1 mg. ^2^ Coxirill® 0.2% (Huvepharma, Sofia, Bulgaria; a.p. diclazuril, 2 mg/kg product; 1 mg diclazuril/kg diet). ^3^ Oxiter 200 BMP (DOX-AL Italia S.p.A., Milan, Italy; active substance oxytetracycline, 200 g/kg product; 7250 mg/kg diet included in feed mixture preparation corresponding to 1450 mg oxytetracycline/kg diet). HE–HP = diet with high digestible energy and high crude protein content (10.9 MJ DE/kg; 159 g CP/kg). HE–LP = diet with high digestible energy and low crude protein content (10.8 MJ DE/kg; 146 g CP/kg). LE–HP = diet with low digestible energy and high crude protein content (9.1 MJ DE/kg; 159 g CP/kg). LE–LP = diet with low digestible energy and low crude protein content (9.3 MJ DE/kg; 142 g CP/kg). F = fattening diet.

**Table 2:** Growth performance of rabbits from 33 to 76 days of age.

	Genotype (Gen)		Post-Weaning Diets (Diet)				*p*-Value					RMSE ^1^
	G	H	HE–HP	HE–LP	LE–HP	LE–LP	Gen	Diet	DE	CP	Gen × Diet	
Rabbits (*n*)	159	164	82	82	79	80						
Live weight (g)												
at 33 days	914	967	933	939	942	950	<0.001	0.775	0.395	0.540	0.963	102
at 64 days	2566	2618	2608	2608	2575	2577	0.006	0.363	0.075	0.979	0.876	197
at 76 days	3100	3120	3091	3093	3120	3137	0.423	0.434	0.116	0.687	0.762	234
Weight gain (g/d)												
33 to 64 days	52.4	54.1	53.8	53.8	52.7	52.8	0.006	0.363	0.075	0.979	0.876	5.2
64 to 76 days	44.5	41.8	40.2 ^a^	40.4 ^a^	45.4 ^b^	46.7 ^b^	0.004	<0.001	<0.001	0.413	0.388	8.1
33 to 76 days	50.2	50.7	50.0	50.0	50.7	51.1	0.428	0.412	0.116	0.687	0.762	4.8
Feed intake (g/d)												
33 to 64 days	154	158	144 ^a^	145 ^a^	170 ^b^	165 ^b^	0.024	<0.001	<0.001	0.141	0.671	14
64 to 76 days	186	178	176 ^a^	176 ^a^	188 ^b^	189 ^b^	0.001	<0.001	<0.001	0.855	0.332	20
33 to 76 days	163	163	153 ^a^	153 ^a^	175 ^b^	171 ^b^	0.878	<0.001	<0.001	0.341	0.665	14
Feed conversion ratio (g/g)												
33 to 64 days	2.95	2.92	2.67 ^a^	2.69 ^a^	3.23 ^c^	3.14 ^b^	0.156	<0.001	<0.001	0.134	0.935	0.16
64 to 76 days	4.22	4.29	4.38 ^b^	4.36 ^b^	4.20 ^ab^	4.09 ^a^	0.247	0.001	<0.001	0.278	0.809	0.54
33 to 76 days	3.25	3.23	3.06 ^a^	3.07 ^a^	3.46 ^b^	3.37 ^b^	0.176	<0.001	<0.001	0.158	0.917	0.18

^1^ Root mean square error. G = Grimaud crossbred rabbits. H = Hyla crossbred rabbits. HE–HP = diet with high digestible energy and high crude protein content (10.9 MJ DE/kg; 159 g CP/kg). HE–LP = diet with high digestible energy and low crude protein content (10.8 MJ DE/kg; 146 g CP/kg). LE–HP = diet with low digestible energy and high crude protein content (9.1 MJ DE/kg; 159 g CP/kg). LE–LP = diet with low digestible energy and low crude protein content (9.3 MJ DE/kg; 142 g CP/kg). DE = Probability of the contrast ((HE–HP + HE–LP)/2) vs. ((LE–HP + LE–LP)/2). CP = Probability of the contrast ((HE–HP + LE–HP)/2) vs. ((HE–LP + LE–LP)/2). ^a,b,c^ Different letters over the means indicate significant differences (*p *< 0.05).

**Table 3:** Faecal apparent digestibility of nutrients and nutritive value (as-fed basis) of the post-weaning diets measured in a digestibility trial from 46 to 50 days of age.

	Genotype (Gen)		Post-Weaning Diets (Diet)				*p*-Value					RMSE ^1^
	G	H	HE–HP	HE–LP	LE–HP	LE–LP	Gen	Diet	DE	CP	Gen × Diet	
Rabbits (*n*)	36	36	18	18	18	18						
Feed intake (g/d)	160	173	160 ^a^	154 ^a^	178 ^b^	175 ^b^	<0.001	<0.001	<0.001	0.187	0.827	14
Dry matter (%)	61.8	61.6	67.2 ^c^	66.3 ^c^	55.8 ^a^	57.5 ^b^	0.550	<0.001	<0.001	0.164	0.449	1.3
Crude protein (%)	76.5	75.6	77.3 ^b^	77.3 ^b^	74.7 ^a^	75.0 ^a^	0.010	<0.001	<0.001	0.757	0.132	1.4
Ether extract (%)	80.2	82.2	83.9 ^b^	83.1 ^b^	79.1 ^a^	78.5 ^a^	<0.001	<0.001	<0.001	0.091	0.101	1.7
aNDF (%)	33.5	34.0	40.0 ^b^	38.1 ^b^	28.2 ^a^	28.8 ^a^	0.368	<0.001	<0.001	0.238	0.773	2.4
ADF (%)	26.0	26.9	32.0 ^b^	30.5 ^b^	21.2 ^a^	22.0 ^a^	0.140	<0.001	<0.001	0.532	0.451	2.5
Lignin (%)	17.6	17.9	24.2 ^b^	21.9 ^b^	11.8 ^a^	13.3 ^a^	0.731	<0.001	<0.001	0.590	0.445	3.4
Gross energy (%)	61.0	60.9	66.6 ^c^	66.0 ^c^	54.8 ^a^	56.4 ^b^	0.804	<0.001	<0.001	0.124	0.518	1.4
Starch (%)	98.8	98.7	98.2 ^a^	98.4 ^a^	99.2 ^b^	99.1 ^b^	0.103	<0.001	<0.001	0.309	0.170	0.2
Digestible protein (DP) (g/kg)	115.8	114.5	122.7	112.7	118.7	106.7	n.d. ^2^	n.d.	n.d.	n.d.	n.d.	n.d.
Digestible energy (DE) (MJ/kg)	10.0	10.0	10.9	10.8	9.1	9.3	n.d.	n.d.	n.d.	n.d.	n.d.	n.d.
DP to DE ratio (g/MJ)	11.6	11.5	11.3	10.5	13.1	11.5	n.d.	n.d.	n.d.	n.d.	n.d.	n.d.

^1^ Root mean square error. ^2^ Not determined. G = Grimaud crossbred rabbits. H = Hyla crossbred rabbits. HE–HP = diet with high digestible energy and high crude protein content (10.9 MJ DE/kg; 159 g CP/kg). HE–LP = diet with high digestible energy and low crude protein content (10.8 MJ DE/kg; 146 g CP/kg). LE–HP = diet with low digestible energy and high crude protein content (9.1 MJ DE/kg; 159 g CP/kg). LE–LP = diet with low digestible energy and low crude protein content (9.3 MJ DE/kg; 142 g CP/kg). DE = Probability of the contrast ((HE–HP + HE–LP)/2) vs. ((LE–HP + LE–LP)/2). CP = Probability of the contrast ((HE–HP + LE–HP)/2) vs. ((HE–LP + LE–LP)/2). ^a,b,c^ Different letters over the means indicate significant differences (*p* < 0.05).

**Table 4:** Faecal apparent digestibility of dietary components and nutritive value (as-fed basis) of the fattening diet (digestibility trial from 72 to 76 days of age).

	Genotype (Gen)		Post-Weaning Diets (Diet)				*p*-Value					RMSE ^1^
	G	H	HE–HP	HE–LP	LE–HP	LE–LP	Gen	Diet	DE	CP	Gen × Diet	
Rabbits (*n*)	16	16	8	8	8	8						
Feed intake (g/d)	162	173	154	165	167	183	0.207	0.123	0.054	0.105	0.651	20
Dry matter (%)	65.9	66.6	66.1	65.9	66.7	66.3	0.276	0.757	0.418	0.567	0.148	1.6
Crude protein (%)	70.8	71.9	70.8	71.4	72.4	70.8	0.045	0.080	0.330	0.290	0.200	1.3
Ether extract (%)	84.4	85.8	85.4 ^b^	84.1 ^a^	85.4 ^b^	85.5 ^b^	<0.001	0.002	0.013	0.030	0.900	0.7
aNDF (%)	35.4	35.9	36.0	34.5	37.6	34.6	0.692	0.164	0.416	0.060	0.324	3.0
ADF (%)	26.3	28.4	27.8	26.3	29.4	26.0	0.117	0.212	0.640	0.070	0.198	3.3
Lignin (%)	13.2	10.2	10.7	10.9	14.1	11.2	0.072	0.365	0.264	0.400	0.681	4.1
Gross energy (%)	65.3	65.8	65.5	65.1	65.9	65.6	0.405	0.815	0.497	0.580	0.174	1.6
Starch (%)	98.7	98.8	98.6	98.8	98.8	98.8	0.010	0.101	0.086	0.206	0.100	0.2
Digestible protein (DP) (g/kg)	106.5	107.6	106.5	106.9	108.4	106.4	n.d.	n.d.	n.d.	n.d.	n.d.	n.d.
Digestible energy (DE) (MJ/kg)	10.6	10.7	10.7	10.6	10.7	10.7	n.d. ^2^	n.d.	n.d.	n.d.	n.d.	n.d.
DP to DE ratio (g/MJ)	10.0	10.1	10.0	10.1	10.1	10.0	n.d.	n.d.	n.d.	n.d.	n.d.	n.d.

^1^ Root mean square error. ^2^ Not determined. G = Grimaud crossbred rabbits. H = Hyla crossbred rabbits. HE–HP = diet with high digestible energy and high crude protein content (10.9 MJ DE/kg; 159 g CP/kg). HE–LP = diet with high digestible energy and low crude protein content (10.8 MJ DE/kg; 146 g CP/kg). LE–HP = diet with low digestible energy and high crude protein content (9.1 MJ DE/kg; 159 g CP/kg). LE–LP = diet with low digestible energy and low crude protein content (9.3 MJ DE/kg; 142 g CP/kg). DE = Probability of the contrast ((HE–HP + HE–LP)/2) vs. ((LE–HP + LE–LP)/2). CP = Probability of the contrast ((HE–HP + LE–HP)/2) vs. ((HE–LP + LE–LP)/2). ^a,b^ Different letters over the means indicate significant differences (*p* < 0.05).

**Table 5:** Caecal fermentative activity in rabbits at 50 days of age.

	Genotype (Gen)		Post-Weaning Diets (Diet)				*p*-Value					RMSE ^1^
	G	H	HE–HP	HE–LP	LE–HP	LE–LP	Gen	Diet	DE	CP	Gen × Diet	
Rabbits (*n*)	24	24	12	12	12	12						
Live weight, LW (g)	1924	1941	1918	1958	1879	1977	0.605	0.135	0.758	0.032	0.775	177
Full gut (% LW)	21.8	22.6	22.2	22.4	22.3	21.8	0.150	0.848	0.622	0.712	0.504	1.9
Full stomach (% LW)	5.7	6.1	5.7	5.8	6.1	5.8	0.034	0.244	0.187	0.538	0.783	0.6
Full caecum (% LW)	7.8	7.5	7.9	7.8	7.5	7.5	0.445	0.693	0.243	0.791	0.653	1.0
Caecal content:												
pH	6.05	6.10	5.98 ^a^	5.99 ^a^	6.18 ^b^	6.14 ^b^	0.441	0.046	0.006	0.175	0.558	0.22
N–NH_3_ (mmol/L)	4.58	6.11	4.39 ^a^	3.98 ^a^	7.50 ^b^	5.51 ^ab^	0.094	0.026	0.010	0.175	0.699	2.9
Total VFA (mmol/L)	54.6	50.5	54.4 ^ab^	59.1 ^b^	44.3 ^a^	52.4 ^ab^	0.157	0.005	0.004	0.028	0.051	10.1
C2 (% mol VFA)	78.5	79.3	77.7	78.5	79.8	79.5	0.410	0.314	0.085	0.824	0.370	3.1
C3 (% mol VFA)	6.3	6.1	6.9	5.5	6.4	6.2	0.888	0.137	0.786	0.064	0.179	1.4
C4 (% mol VFA)	13.9	13.1	14.1	14.4	12.5	12.9	0.237	0.126	0.023	0.558	0.854	2.4
C5 (% mol VFA)	0.9	0.8	0.8	0.8	0.9	0.8	0.612	0.962	0.940	0.627	0.060	0.4
C3/C4 ratio	0.48	0.49	0.49	0.42	0.51	0.50	0.528	0.306	0.190	0.277	0.109	0.15

^1^ Root mean square error. G = Grimaud crossbred rabbits. H = Hyla crossbred rabbits. HE–HP = diet with high digestible energy and high crude protein content (10.9 MJ DE/kg; 159 g CP/kg). HE–LP = diet with high digestible energy and low crude protein content (10.8 MJ DE/kg; 146 g CP/kg). LE–HP = diet with low digestible energy and high crude protein content (9.1 MJ DE/kg; 159 g CP/kg). LE–LP = diet with low digestible energy and low crude protein content (9.3 MJ DE/kg; 142 g CP/kg). DE = Probability of the contrast ((HE–HP + HE–LP)/2) vs. ((LE–HP + LE–LP)/2). CP = Probability of the contrast ((HE–HP + LE–HP)/2) vs. ((HE–LP + LE–LP)/2). ^a,b^ Different letters over the means indicate significant differences (*p* < 0.05).

**Table 6:** Live weight, carcass yield, carcass dissection and meat quality of rabbits slaughtered at 77 days of age.

	Genotype (Gen)		Post-Weaning Diets (Diet)				*p*-Value					RMSE ^1^
	G	H	HE–HP	HE–LP	LE–HP	LE–LP	Gen	Diet	DE	CP	Gen × Diet	
Rabbits (*n*)	80	80	40	40	40	40						
Live weight at farm ^2^ (g)	3085	3098	3074	3080	3102	3110	0.584	0.613	0.191	0.761	0.678	225
Live weight at slaughter, SW (g)	2983	2990	2977	2976	2989	3003	0.749	0.793	0.374	0.753	0.767	217
Transport losses (%)	3.30	3.49	3.12 ^a^	3.36 ^ab^	3.65 ^b^	3.46 ^ab^	0.154	0.026	0.012	0.846	0.183	1.14
Chilled carcass weight, CC (g)	1879	1824	1840	1842	1856	1866	<0.001	0.531	0.163	0.691	0.872	145
Carcass yield (% SW)	63.0	61.0	61.8	61.9	62.1	62.1	<0.001	0.409	0.101	0.773	0.695	1.4
Full digestive tract (% LW)	16.1	17.7	16.7	16.9	17.1	16.9	<0.001	0.624	0.310	0.863	0.700	1.5
Other wastes ^3^ (% LW)	20.9	21.5	21.6 ^b^	21.3 ^ab^	20.9 ^a^	21.1 ^ab^	0.001	0.014	0.005	0.522	0.859	1.1
Head (% CC)	7.94	8.10	8.10	7.97	8.10	7.91	0.142	0.444	0.780	0.112	0.866	0.65
Liver (% CC)	4.20	4.29	4.23	4.17	4.21	4.36	0.406	0.594	0.423	0.675	0.240	0.66
Reference carcass, RC (g)	1593	1551	1555	1570	1573	1591	0.020	0.515	0.255	0.326	0.989	118
Dissectible fat (% RC)	2.76	3.14	2.86	3.20	2.77	2.96	0.006	0.145	0.226	0.056	0.053	0.63
*longissimus lumborum* (% RC)	12.3	12.3	12.1	12.4	12.7	12.1	0.984	0.155	0.601	0.497	0.667	1.0
Hind legs (% RC)	33.1	32.8	32.9	32.6	33.3	33.1	0.176	0.081	0.018	0.303	0.970	1.0
Left hind leg muscle/bone ratio	6.33	6.11	5.98	6.19	6.36	6.34	0.146	0.250	0.078	0.524	0.809	0.66
*longissimus lumborum* traits:												
pH	5.64	5.60	5.62	5.63	5.60	5.63	0.055	0.574	0.473	0.259	0.114	0.10
L*	53.4	54.9	54.3	54.0	54.4	53.8	0.010	0.235	0.332	0.258	0.258	2.8
a*	−1.99	−2.19	−2.14	−2.04	−2.16	−2.02	0.342	0.286	0.523	0.324	0.324	0.85
b*	0.69	0.76	0.65	0.80	0.72	0.73	0.295	0.887	0.432	0.285	0.285	2.33
Thawing losses (%)	8.0	8.4	7.5	8.0	8.6	8.7	0.286	0.115	0.280	0.388	0.503	1.8
Cooking losses (%)	30.2	30.4	30.2	30.2	30.2	30.7	0.540	0.482	0.445	0.355	0.547	1.2
Shear force (kg/g)	3.92	4.17	3.99	3.91	4.15	4.11	0.156	0.771	0.323	0.735	0.668	0.79

^1^ Root mean square error. ^2^ Live weight measured after 4 hours of fasting. ^3^ Skin, distal part of fore and hind legs, blood. G = Grimaud crossbred rabbits. H = Hyla crossbred rabbits. HE–HP = diet with high digestible energy and high crude protein content (10.9 MJ DE/kg; 159 g CP/kg). HE–LP = diet with high digestible energy and low crude protein content (10.8 MJ DE/kg; 146 g CP/kg). LE–HP = diet with low digestible energy and high crude protein content (9.1 MJ DE/kg; 159 g CP/kg). LE–LP = diet with low digestible energy and low crude protein content (9.3 MJ DE/kg; 142 g CP/kg). DE = Probability of the contrast ((HE–HP + HE–LP)/2) vs. ((LE–HP + LE–LP)/2). CP = Probability of the contrast ((HE–HP + LE–HP)/2) vs. ((HE–LP + LE–LP)/2). ^a,b^ Different letters over the means indicate significant differences (*p* < 0.05).

## Data Availability

The data presented in this study are available on request from the corresponding author. The data are not publicly available due to privacy restriction (animals and diets provided by private enterprises).
